# Delayed Awakening Following General Anesthesia in a 74-Year-Old Female Patient: An Interplay of Physiological and Psychological Factors

**DOI:** 10.7759/cureus.96646

**Published:** 2025-11-12

**Authors:** Lin Ba

**Affiliations:** 1 Anesthesiology, Binzhou People's Hospital, Binzhou, CHN

**Keywords:** case report, delayed awakening, emergence delirium, geriatric anesthesia, personality traits, postoperative agitation, preoperative anxiety, psychological factors

## Abstract

Delayed awakening and postoperative agitation pose significant challenges in geriatric anesthesia. This report discusses a 74-year-old female with hypertension and notable preoperative anxiety who underwent laser ablation of a laryngeal tumor under general anesthesia. Despite a hemodynamically stable 1.5-hour procedure, the patient remained unresponsive for over two hours and subsequently developed significant agitation, scoring +2 to +3 on the Richmond Agitation-Sedation Scale. Arterial blood gas analyses were unremarkable, and common pharmacological or metabolic causes were excluded. She spontaneously recovered after approximately three hours, with her family reporting a nearly identical episode two years prior alongside a history of a stubborn temperament, suggesting a potential psychological component. This case highlights that delayed awakening in the elderly can be multifactorial, where psychological predispositions may amplify pharmacological effects. It advocates for integrating psychological assessment into preoperative planning for vulnerable older adults and calls for more research into personalized anesthesia protocols addressing both physiological and mental health factors.

## Introduction

Anesthesia-related complications, particularly delayed awakening, present a significant challenge in perioperative care, especially among older adults with pre-existing medical and psychological conditions [1]. Defined by prolonged unresponsiveness, delayed awakening is frequently accompanied by agitation, which can be exacerbated by underlying psychological factors such as anxiety or specific personality traits [2]. The incidence of these events is substantially higher in elderly patients, with key risk factors including advanced age, comorbidities, and altered pharmacodynamics [3,4].

The case of this 74-year-old female patient is highly instructive. She developed pronounced delayed awakening with marked agitation following an otherwise uncomplicated surgical procedure. Her history of hypertension, combined with a previous similar episode and notable behavioral characteristics, illustrates the multifactorial etiology of such complications [5].

## Case presentation

The patient was scheduled for laryngeal mass excision under general anesthesia. She had a 30-year history of hypertension, controlled with daily telmisartan 40 mg. Preoperatively, she was alert but displayed evident anxiety and tension; no formal psychological screening was conducted.

General anesthesia was induced with the sequential administration of midazolam 1 mg, fentanyl 0.3 mg, propofol 100 mg, and rocuronium 35mg. It was maintained with sevoflurane (end-tidal concentration 1.5-2.0%) and a remifentanil infusion (0.1-0.2 mcg/kg/min). Depth of anesthesia was guided by Bispectral Index (BIS) monitoring, which was maintained between 50 and 60 throughout the procedure. The 1.5-hour procedure was uneventful, with stable hemodynamics (mean arterial pressure maintained between 70-90 mmHg, heart rate between 60-85 beats per minute, and no episodes of persistent hypotension or hypertension) and adequate oxygenation. Neuromuscular blockade was fully reversed with sugammadex prior to emergence. As part of the structured emergence protocol, flumazenil 1 mg was administered intravenously to antagonize the midazolam; however, the patient remained unresponsive. Upon achieving an end-tidal sevoflurane concentration of 0% and with the BIS value sustained above 90 for approximately 10 minutes, the trachea was extubated.

Despite the comprehensive reversal of anesthetic agents, the patient remained unresponsive to verbal stimuli for over two hours in the post-anesthesia care unit (PACU). From admission, she had fully restored muscle strength, was normothermic, and exhibited persistent agitation. Notably, her pupils were normal in size and reactive to light. Neurological examination confirmed normal pupillary reflexes and appropriate withdrawal to painful stimuli. Her agitation was scored at +2 to +3 on the Richmond Agitation-Sedation Scale (RASS) [6].

To elucidate the cause of delayed awakening, two arterial blood gas analyses were performed postoperatively. The first analysis (Table 1), conducted 30 minutes after surgery, revealed a mild respiratory acidosis (pH 7.284, pCO₂ 50.4 mmHg), likely reflecting residual anesthetic-induced hypoventilation at the time of sampling. The follow-up analysis (Table 2), obtained one hour postoperatively after patient stimulation, confirmed the resolution of this imbalance, with all key parameters returning to normal ranges, thus ruling out a persistent metabolic or respiratory derangement.

**Table 1:** Arterial blood gas analysis results half an hour after surgery This table shows the arterial blood gas results half an hour after surgery. pCO₂, partial pressure of carbon dioxide; pO₂, partial pressure of oxygen; SO₂, oxygen saturation; FO₂Hb, fraction of oxyhemoglobin; FCOHb, fraction of carboxyhemoglobin; FMetHb, fraction of methemoglobin; HCO₃⁻, bicarbonate; FiO₂, fraction of inspired oxygen.

Variable	Result	Reference range
pH	7.284	7.35-7.45
pCO₂	50.4	32-48 mmHg
pO₂	62.1	83-108 mmHg
Total hemoglobin	146	135-175 g/L
SO₂	91.4	95-99%
F0_2_Hb	89.1	94-98%
FCOHb	1.8	0.5-1.5%
FMetHb	0.8	0.0-1.5%
Sodium	143.7	136-146 mmol/L
Potassium	3.47	3.4-4.5 mmol/L
Chloride	111.2	98-106 mmol/L
Calcium	1.196	1.15-1.29 mmol/L
Lactate	1.5	0.5-1.6 mmol/L
HCO₃⁻	23.4	21-28 mmol/L
FiO₂	21	21-100%
Patient’s temperature	37	36-37°C

**Table 2:** Arterial blood gas analysis results one hour after surgery This table shows the arterial blood gas results one hour after surgery. pCO₂, partial pressure of carbon dioxide; pO₂, partial pressure of oxygen; SO₂, oxygen saturation; FO₂Hb, fraction of oxyhemoglobin; FCOHb, fraction of carboxyhemoglobin; FMetHb, fraction of methemoglobin; HCO₃⁻, bicarbonate; FiO₂, fraction of inspired oxygen.

Variable	Result	Reference range
pH	7.302	7.35-7.45
pCO₂	45.8	32-48 mmHg
pO₂	68.4	83-108 mmHg
Total hemoglobin	146	135-175 g/L
SO₂	94	95-99%
F0_2_Hb	94	94-98%
FCOHb	1.8	0.5-1.5%
FMetHb	0.8	0.0-1.5%
Sodium	140.9	136-146 mmol/L
Potassium	3.6	3.4-4.5 mmol/L
Chloride	110.5	98-106 mmol/L
Calcium	1.177	1.15-1.29 mmol/L
Lactate	1.4	0.5-1.6 mmol/L
HCO₃⁻	22.1	21-28 mmol/L
FiO₂	21	21-100%
Patient’s temperature	37	36-37°C

These serial blood gas results, combined with the failure of flumazenil to restore consciousness and the confirmed reversal of neuromuscular blockade, collectively excluded common physiological causes such as residual sedation, neuromuscular blockade, hypothermia, drug overdose, or acute neurological injury. In the absence of an identifiable physiological cause, she was transferred to the ward. Approximately three hours post-surgery, she spontaneously regained full consciousness with no recall of the event. Her family reported a nearly identical episode two years earlier and specifically denied any family history of similar anesthetic complications. They described her as having a "generally stubborn and anxious temperament."

## Discussion

This case of profound delayed awakening and agitation in a 74-year-old woman offers valuable insight into the challenges of geriatric anesthesia. The recurrence of an identical postoperative pattern, in the absence of identifiable pharmacological or metabolic causes, strongly implicates intrinsic patient factors, particularly psychological makeup [2,5].

Age-related physiological changes-such as declines in hepatic and renal function-predispose older adults to prolonged effects of anesthetic agents like sevoflurane and remifentanil [3,4]. However, the extent and duration of unresponsiveness and agitation observed here exceed what would be expected from pharmacokinetics alone. Furthermore, the failure of flumazenil to restore consciousness, despite its administration as part of a structured emergence protocol, along with the confirmed reversal of neuromuscular blockade, makes residual drug effects from benzodiazepines or neuromuscular blocking agents a highly unlikely primary cause [7].

Thus, the role of psychological factors becomes paramount. The patient's significant preoperative anxiety and her family's description of a "stubborn temperament" suggest that underlying psychological traits substantially influenced her emergence profile. Preoperative anxiety is a well-established risk factor for postoperative delirium and agitation [8,9]. In susceptible individuals, emergence from anesthesia may unmask or amplify latent anxiety or inflexible personality traits, leading to behaviors that can be misinterpreted as delayed awakening [5,10].

Beyond psychological composition, individual variations in drug sensitivity, particularly to opioids, and potential genetic polymorphisms affecting drug metabolism or neurotransmitter systems may also contribute to such atypical emergence phenomena [11,12]. While no other opioids besides remifentanil and a single induction dose of fentanyl were used in this case, the potential role of these factors warrants consideration in the multifactorial analysis of delayed awakening.

This case highlights a critical gap in preoperative evaluation: while medical comorbidities are routinely assessed, psychological screening is often overlooked. The use of brief, validated instruments such as the Hospital Anxiety and Depression Scale (HADS) could help identify at-risk patients [9,13]. For those with relevant histories or traits, anesthetic management can be individualized-for example, through EEG-guided anesthesia to reduce drug dosing [14], avoidance of benzodiazepines, and early implementation of non-pharmacologic calming measures.

A key limitation of this report is the retrospective attribution to psychological factors in the absence of formal preoperative assessment. The lack of baseline cognitive or anxiety scores precludes objective characterization of her mental state. Nevertheless, the recurrent, nearly identical clinical picture supports this interpretation.

## Conclusions

This case illustrates that delayed awakening in older adults may represent a “final common pathway” for diverse etiologies, including psychological predisposition. It emphasizes the importance of a holistic, patient-centered approach in anesthesiology - one that integrates preoperative psychological screening into geriatric assessment and tailors anesthetic plans to a comprehensive risk profile that includes mental health. Further research is essential to clarify the complex relationships among personality, anxiety, and emergence from anesthesia in the aging population.
